# Five recommendations to advance implementation science for humanitarian settings: the next frontier of humanitarian research

**DOI:** 10.1186/s13031-024-00597-2

**Published:** 2024-05-28

**Authors:** Kathryn Falb, Sheree Kullenberg, Christina T Yuan, Alexandra Blackwell

**Affiliations:** 1 Department of International Health, Johns Hopkins Bloomberg School of Public Health, Baltimore, MD USA; 2 Airbel Impact Lab, International Rescue Committee, New York City, NY USA; 3 Department of Health Policy and Management, Johns Hopkins Bloomberg School of Public Health, Baltimore, MD USA; 4 Department of Social Policy and Intervention, University of Oxford, Oxford, UK 32 Wellington Square, OX1 2ER

**Keywords:** Implementation science, Humanitarian, Conflict, Evidence-based interventions

## Abstract

Challenges in delivering evidence-based programming in humanitarian crises require new strategies to enhance implementation science for better decision-making. A recent scoping review highlights the scarcity of peer-reviewed studies on implementation in conflict zones. In this commentary, we build on this scoping review and make five recommendations for advancing implementation science for humanitarian settings. These include (1) expanding existing frameworks and tailoring them to humanitarian dynamics, (2) utilizing hybrid study designs for effectiveness-implementation studies, (3) testing implementation strategies, (4) leveraging recent methodological advancements in social and data science, and (5) enhancing training and community engagement. These approaches aim to address gaps in understanding intervention effectiveness, scale, sustainability, and equity in humanitarian settings. Integrating implementation science into humanitarian research is essential for informed decision-making and improving outcomes for affected populations.

## Background

Delivering evidence-based programming in humanitarian crises grappling with war, climate change, and disaster is a formidable challenge. To support this goal, rigorous impact evaluations are increasingly being implemented to deliver better aid from evaluating the impact of cash transfers [1], promoting better education for children [2], advancing mental health and psychosocial programming for people affected by disaster [3], and improving the safety and wellbeing of women and children in conflict-affected zones [4, 5]. Methodologies have also advanced substantially such that studies can be pre-positioned for acute crises to promote the effectiveness of anticipatory action or find creative approaches to build comparison groups that do not delay receipt of aid, but rather ethically exploit organic programmatic delivery cycles and targeting procedures [6] or use propensity score matching approaches [7]. Nonetheless, while the generation of rigorous evidence of impact has increased, the ability to reach scale, sustainability, and equity across settings within humanitarian programming has been suboptimal.

## Identifying and addressing gaps in implementation science for humanitarian settings

To address this challenge, the recently published scoping review conducted by Leresche and colleagues (2023), focuses on how implementation science or operational research may be used to improve evidence-based decision-making by humanitarian practitioners [8]. The authors find only 22 studies across 34 countries experiencing conflict that provide data on implementation or operational research processes. To assess the implementation of new interventions, studies in the review employed mixed methods, with the vast majority employing qualitative methods (*n* = 21) and others using a mix of routine data such as retrospective records reviews (*n* = 9) or monitoring data (*n* = 10). Only a few studies used evaluative methods such as a cluster randomised trial (*n* = 1) or cost analysis (*n* = 2) to document implementation. The authors acknowledge that a major limitation of the scoping review is the exclusion of studies that were not peer reviewed. Indeed, in humanitarian settings, frontline providers collect valuable data to track progress on activities, record outputs, and monitor for potential adverse effects of interventions. These data are often synthesised into reports and lessons learned documents and sometimes used to refine implementation approaches in the short term. However, this information is rarely captured and stored systematically and almost never peer reviewed. Moreover, the tools to collect these data are not informed by implementation science frameworks. As such, there remains a gap in the approaches used by practitioners and researchers to systematically document implementation processes. This gap impedes our ability to build evidence on humanitarian interventions. The authors conclude by proposing an important contribution regarding an adapted normalization process theory that highlights the role of frontline workers and communities in the implementation of evidence-based practices.

Based on our collective experience evaluating and implementing research in humanitarian settings, we are unsurprised with the limited number of studies included in the review. We further hypothesize that challenges related to evidence-based decision-making could also arise because there is a lack of implementation science frameworks, models, or theories that account for humanitarian dynamics and that can be readily integrated into routine data collection efforts or (quasi) experimental designs examining effectiveness of programming. The lack of appropriate frameworks and their application diminish the ability to understand how, why, and under what circumstances interventions are effective—and ultimately decrease the humanitarian field’s ability to integrate new knowledge into programming approaches. Therefore, to improve the evidence base such that humanitarian action can achieve impact, scale, sustainability, and equity, we recommend the following research avenues in unstable humanitarian settings:

### Expand current implementation science frameworks to more holistically address humanitarian crises factors that influence program impact, scale, sustainability, and equity

The findings of the scoping review indicated that the conflict context itself interrupts the uptake of evidence-based practices, and conflict-related disruptions require implementing actors to constantly adapt and negotiate to sustain the provision of humanitarian response. The authors identified the need for interventions to be flexible to account for these disruptions and balance effectiveness with feasibility, acceptability, and validity of interventions for both frontline service providers and communities. Considering the recurrent operational, organizational, and security hurdles that frequently impede or restrict research and implementation in conflict zones, it is imperative to evaluate the impact of the conflict context on the implementation process itself. However, current implementation frameworks are suboptimal in accounting for conflict-related disruptions and how these events also influence service providers and communities.

As such, several existing frameworks could be expanded to incorporate some of these considerations. For example, the RE-AIM (Reach, Effectiveness, Adoption, Implementation, and Maintenance) framework evaluates multiple domains of implementation outcomes and their associated social behavioural outcomes [9, 10]. Though it has not been used in humanitarian settings, the RE-AIM framework has been applied to the evaluation of community-based health interventions [11, 12] and to assess stakeholder engagement as an outcome of feasibility [13] which could be beneficial for assessing the engagement of implementing actors early in implementation of humanitarian interventions. Determinant frameworks such as EPIS (Exploration, Preparation, Implementation, and Sustainment) and the Consolidated Framework for Implementation Research (CFIR) that consider the outer and inner context when examining implementation across multiple domains and stages could be used to assess the environment of the humanitarian context, specifically [14, 15]. While the EPIS framework has not yet been applied in a humanitarian setting, its inclusion of the outer context as a dynamic component of implementation for complex public service interventions could serve as a model for examining the influence of conflict-related factors on implementation [16], as well as their effects on frontline service providers and communities [17]. We contend that the Dynamic Sustainability Framework, with its particular attention to understanding how changes in context may influence sustainment of interventions, may be particularly advantageous to explore within studies and programs that are continually refined over time and place [18]. 

Leresche and colleagues’ Extended Normalization Process Theory (ENPT) proposal offers a promising avenue through which to incorporate humanitarian frontline worker and community insights into these frameworks. Building on determinant frameworks, the ENPT theorises how the dynamic elements of a context play a role in shaping service providers’ opportunity and potential for implementation of an intervention [19]. To apply this and other implementation science frameworks to humanitarian action, efforts are needed to operationalise the conflict context to adequately account for its influence on the agency and capabilities of service providers as they negotiate the many challenges of the contexts in which they live and work; for example, measuring disruptions, displacement, policy shifts, or constraints on resources and how these interact with the feasibility of an intervention.

### Increase the use of effectiveness-implementation hybrid designs in experimental programming

Three types of hybrid designs have been used to examine the impact of interventions alongside their implementation [20]. Type 1 includes testing effectiveness of an intervention while gathering implementation data; Type 2 includes jointly testing effectiveness and implementation strategies, while Type 3 includes testing an implementation strategy while observing effectiveness outcomes. In experimental studies, researchers should be gathering data and publishing inferences on implementation alongside of their effectiveness to link intervention outcomes with implementation factors. To date, the limitations of the evidence base on implementation of humanitarian interventions stem largely from the lack of peer-reviewed studies examining implementation outcomes such as adoption, fidelity, sustainability, etc. Within humanitarian action where flexibility in intervention modalities, delivery, and resourcing is key, it is no longer sufficient to publish conclusions on intervention efficacy, alone. Strengthening these effectiveness-implementation designs can be achieved through the embedding of implementation tools into ongoing trials based on clear conceptual frameworks. Conversely, approaches used by frontline providers to produce practice-based knowledge on implementation can be strengthened through methodological advancements that enable the systematic documentation of implementation outcomes even in routine assessments. In doing so, these data can be used more effectively in answering implementation science questions.

Across all effectiveness-implementation hybrid designs, mixed methods approaches should be used to combine quantitative and qualitative data on both behavioural and implementation outcomes. For example, participatory qualitative techniques with frontline providers and communities such as rapid appraisal, concept mapping, and process analyses can be used to not only explore whether statistical findings reflect community perceptions of outcomes but also whether null or negative findings are related to design or implementation failure [21]. Mixed methods approaches can also enhance flexibility in research design and dissemination for different audiences, which is valuable for advancing humanitarian research and action.

### Systematically test implementation strategies and core components within humanitarian programming

Partnering with local actors to deliver programming with supportive coaching, training, or other quality assurance processes is increasingly practiced within the larger humanitarian architecture. Simultaneously, delivery strategies have evolved to enable rapid response, such as providing phone-based information services or emergency cash transfers to reach more people in less time. Other new strategies focus on increasing the quality of the humanitarian workforce, including task shifting [22, 23], varying financial incentives, or other strategies such as training programmes for health providers supported by technology and artificial intelligence. Developed based on need rather than evidence, these implementation strategies must be tested so that leaders are able to make informed decisions about programming models, delivery mechanisms, and human resourcing while also understanding the impact these decisions may have on effectiveness, reach, sustainability, equity, and cost efficiency.

Relatedly, testing implementation strategies should occur alongside research that seeks to understand the core components and mechanisms of change so that interventions are more easily transferable across contexts and types of emergencies (e.g., protracted or acute, camp or urban setting, etc.) [24]. Transferability of evidence-based interventions to humanitarian programming assumes that experimental, precision-based treatments are appropriate in settings where frontline providers must continually and quickly adapt to meet the needs of populations in fragile contexts. Implementation science must be expanded to consider how interventions can be designed and evaluated to allow for flexibility across implementation domains. Complementary research designs such as the Multiphase Optimization Strategy [25] or A/B testing may be particularly well-suited for humanitarian contexts where identifying core intervention components can improve flexibility and increase the plasticity of implementation [19], allowing for scaling and sustainment within humanitarian systems. Practitioners are more likely to be open to this type of research given its increased contextual relevance and usefulness in making critical design and implementation decisions.

### Leverage methodological advancements to support implementation science in humanitarian settings

Implementation science frameworks can be tested and strengthened through methodological advancements that will improve the quality and usability of evidence in humanitarian settings. For example, the incorporation of systems science could be used to model complex and interrelated factors to improve decision-making for humanitarian action [26]. Through the application of systems thinking tools, researchers could integrate practice-based knowledge in the conceptualisation and testing of causal relationships and underlying structures within intervention systems. Additionally, advancements in machine learning and data-driven artificial intelligence could be applied to implementation science frameworks to enable anticipatory humanitarian action and improve accountability and effectiveness of interventions for crisis-affected populations [27]. These techniques could also be applied to the analysis of non-experimental data to strengthen insights from routinely collected data, enabling better prediction of intervention effect. Combining these approaches with participatory methods with frontline workers and affected communities could improve the rigor of data while upholding considerations of equity and ethics and participation in the development and execution of these new approaches [28]. 

While such recent methodological advancements in social and data sciences spark innovation, at the most basic level, the dearth of systematic approaches for measuring, collecting, and analysing data on implementation substantially impedes our understanding of how different implementation domains support or hinder interventions from achieving their intended outcomes for affected populations. Few standardised tools for measuring implementation factors exist [29–31] and there is little understanding of the psychometric properties of existing tools for implementation evaluations. Measurement must be advanced to improve the usefulness and relevancy of implementation outcomes in real-world settings [32], but particularly in humanitarian settings where feasibility and context-validity of measures are essential. Improvements in data systems could also enhance multilevel modelling of complex interventions within dynamic humanitarian systems using some of the advanced data science techniques mentioned above [33]. 

### Advance implementation science training approaches and systematize practice-based learning and community engagement in collaboration with researchers in humanitarian settings

Training programs for researchers from and living within humanitarian settings should be developed and invested in to elevate their leadership and advance contextually grounded implementation insights. Efforts to systematize practice-based knowledge from practitioners and meaningfully engage communities should also be undertaken to promote their role in the design, execution, and dissemination of humanitarian research [34]. As Leresche and colleagues identify, service providers and communities are essential to not only designing and adapting interventions but also for informing implementation and compensating for conflict-related disruptions. The successful completion of both research and implementation requires collaboration to overcome the numerous challenges that impede programming in humanitarian settings. Action- and practice-based approaches can promote co-design and community ownership and supervision through linkages with local provider systems that prevent the imposition of externally-designed interventions and impractical research designs while also reducing risk of harm [35, 36]. These approaches also promote the generation of evidence for action, whereby research is disseminated to a target audience with concrete programmatic steps and is rapidly used to inform implementation decisions [35]. 

## Conclusion

While the humanitarian field must continue to build the experimental evidence base to understand what works to support populations affected by emergencies, it is critical that humanitarian research goes beyond effectiveness studies to systematically incorporate implementation science to better inform decision-making and strategies to reach impact, scale, sustainability, and equity. This can be achieved by advancing implementation science frameworks to account for time-varying macro-and micro-factors that typify humanitarian settings, elevating the use of effectiveness-implementation hybrid designs, testing implementation strategies and core components, leveraging methodological advancements from other fields, and supporting the training and leadership of researchers, practitioners, and communities living within humanitarian settings. This next frontier of humanitarian research should be supported by appropriate donor investments that recognize the utility of implementation science as part of rigorous research and learning [37]. Only then, can we bridge the gap from effectiveness studies to action and accountability to improve the lives and wellbeing of populations affected by war and disaster.

## Data Availability

Not applicable.
